# Adverse skin reactions among health care workers using face personal protective equipment during the coronavirus disease 2019 pandemic: A cross‐sectional survey of six hospitals in Denmark

**DOI:** 10.1111/cod.14022

**Published:** 2021-12-27

**Authors:** Jette G. Skiveren, Malene F. Ryborg, Britt Nilausen, Susan Bermark, Peter A. Philipsen

**Affiliations:** ^1^ Department of Dermatology and Copenhagen Wound Healing Centre Bispebjerg University Hospital Copenhagen Denmark

**Keywords:** adverse skin reaction, allergic contact dermatitis, health care workers, irritant dermatitis, mask‐related dermatitis, occupational dermatitis, personal protective equipment, skin type

## Abstract

**Background:**

Health care workers (HCWs) report frequent adverse skin reactions (ASRs) due to face personal protective equipment (F‐PPE) use during the coronavirus disease 2019 (COVID‐19) pandemic.

**Objectives:**

To describe self‐reported ASRs among HCWs using F‐PPE; investigate background factors, such as chronic skin diseases and skin types (dry, oily, combination, sensitive), and determine whether HCWs took preventive methods against ASRs.

**Methods:**

An online questionnaire was distributed to 22 993 HCWs at hospitals.

**Results:**

The prevalence of ASRs was 61.9% based on 10 287 responders. Different types of F‐PPE caused different reactions. The most common ASRs from surgical masks were spots and pimples (37.2%) and from FFP3 masks was red and irritated skin (27.3%). A significantly higher proportion of HCWs with chronic skin diseases had ASRs (71.6%) than those without chronic skin diseases (59.7%) (*P* < .001). Some skin types were more prone to ASRs (sensitive skin [78.8%] vs dry skin [54.3%]; *P* = .001). HCWs using F‐PPE for >6 hours versus <3 hours per day had a four times higher ASR risk (*P* = <.001). Nearly all HCWs used preventive and/or counteractive methods (94.2%).

**Conclusions:**

It is important to consider background factors, such as chronic skin diseases and skin types, to prevent and counteract ASRs due to F‐PPE use.

## Graphical abstract

- Daily long‐term use of face protective equipment increased the risk of adverse skin reaction by four times.
- Health care workers (HCWs) with chronic skin diseases had significantly more skin reactions than those without. Sensitive skin types were more prone to skin reactions.
- The skin reactions were higher in HCWs in intensive care units than HCWs in inpatient clinics.

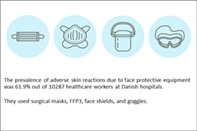

## 1 INTRODUCTION

Since late 2019, coronavirus disease 2019 (COVID‐19) has spread rapidly worldwide. Due to the high transmission rate, health care workers (HCWs) are required to wear face personal protective equipment (F‐PPE) for several hours each working day.^1^, ^2^

The skin is the body's defense against the environment; it is constantly exposed to external factors. The protective function of the skin is altered when it is constantly aggravated by the continuous use of F‐PPE. The skin is repeatedly subjected to physical and chemical factors, such as friction, tension, and pressure, as well as moisture, humidity, and heat.^3^, ^4^ This is associated with the development of friction injuries, skin breakdown, and pressure ulcers.^3^

During the severe acute respiratory syndrome epidemic in 2003, N95 mask–related adverse skin reactions (ASRs) among Singaporean HCWs included acne, facial pruritus, and rash.^5^ In the current COVID‐19 pandemic, cross‐sectional surveys and several case reports have described ASRs related to the use of F‐PPE.^1^, ^2^, ^6^, ^7^, ^8^, ^9^, ^10^, ^11^, ^12^, ^13^, ^14^, ^15^ The most frequently reported ASRs were dry, itchy, red, scaly, macerated, and/or painful skin, as well as pimples, buds, fissures, scratch marks, pressure marks, and ulcers. A systematic review highlighted cases of occupational dermatitis associated with F‐PPE and pointed out the need for well‐designed studies to better understand the incidence and management of mask‐related dermatitis.^6^

Studies have described a correlation between skin types and dermatological diseases, for example, acne and atopic dermatitis.^16^, ^17^, ^18^ Additional studies have shown that prolonged use of F‐PPE can result in the worsening of chronic skin diseases, such as acne or atopic dermatitis.^2^, ^15^

In this study, we examined the use of F‐PPE, including surgical masks, particulate respirators (FFP3/N95), goggles, and facial shields. The use of different types of F‐PPE was based on the recommendations of the authorities. For example, surgical masks were recommended for use in all public indoor areas, whereas FFP3 were prescribed for specific situations for example, caring for patients with COVID‐19.

The objectives in this study were to describe self‐reported ASRs among HCWs using F‐PPE during the COVID‐19 pandemic; investigate background factors, such as chronic skin diseases and skin types (dry, oily, combination, sensitive); and determine whether HCWs had taken preventive methods against ASRs.

## 2 METHODS

### 2.1 Ethical considerations

The study was approved by the Danish Data Protection Agency (approval number: P‐2020‐621) and the crisis management leaders of the Capital Region of Denmark. The research was conducted in accordance with the Declaration of Helsinki. Respondents were informed in writing about the study's objectives and their rights, including anonymity and the freedom to participate voluntarily.

### 2.2 Survey and participants

The survey was designed as an online questionnaire (SurveyXact; Rambøll, Oslo, Norway). The questionnaire was developed based on literature review and it included questions on ASRs, chronic skin diseases, allergies, skin types, risk factors, prevention, and treatments.^5^, ^6^, ^16^, ^17^, ^18^ The survey questions related to ASRs that occurred between October 1, 2020 and February 28, 2021. The questionnaire was emailed to HCWs at six hospitals in the Capital Region of Denmark in February 2021. Reminders were sent to non‐respondents after 1 and 3 weeks.

**FIGURE 1:**
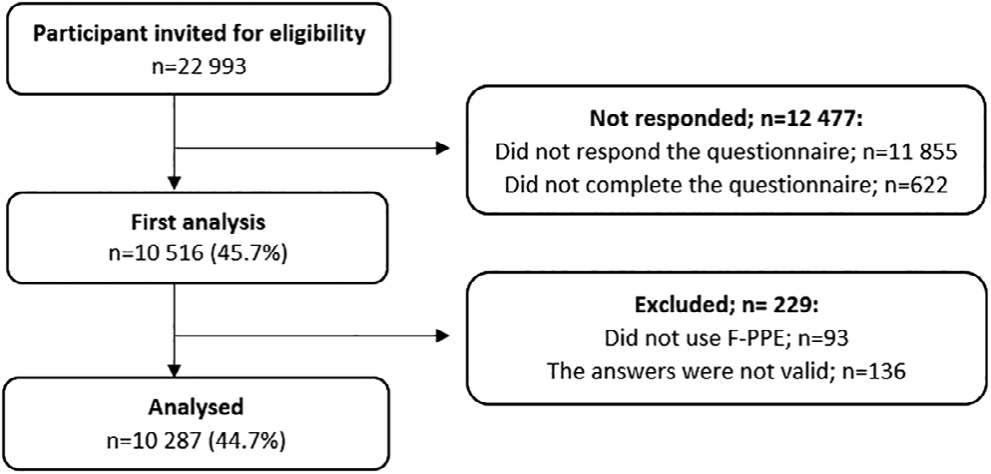
Flow chart of participants and drop‐outs in the study of adverse skin reactions among health care workers at six Danish hospitals

In total, the questionnaire was sent to 22 993 HCWs, of whom 11 855 did not respond, and 622 had an incomplete response (Figure 1). In the analysis of the respondent group (n = 10 516; 45.7%), we excluded HCWs whose answers were invalid, such as those who worked for >168 hours a week, were aged >100 or <18 years old (n = 136), and who did not use F‐PPE (n = 93). If participants reported ASRs, they were reported as having ASRs related to the use of one or more types of F‐PPE.

### 2.3 Statistical analyses

Chi‐square tests were used to investigate associations between categorical variables. Analysis of variance was used to test differences in age and working hours. Binary logistic regression was used for multiple variable analysis of the risk for ASRs. A *P*‐value of <.05 was considered statistically significant. Statistical analyses were performed using SPSS Statistics for Windows (version 25.0; IBM Corporation, Armonk, New York).

## 3 RESULTS

The analyzed population comprised 10 287 HCWs (44.7% of 22 993 invited participants) employed at six hospitals in the Capital Region in Denmark. The population consisted of 8854 women, and 1433 men, with a mean age of 44.8 years, and included nurses, physicians, nursing assistants, physiotherapists, radiographers, midwives, and occupational therapists (Table 1). The HCWs were associated with inpatient care (42.3%), outpatient clinic including radiotherapy (36.6%), operating theatre (7.7%), intensive care unit (7.0%), department of anesthesiology (4.9%), or recovery room (1.7%) (Table 2).

**TABLE 1:** Professions and background of study participants, n (%)

						Use of F‐PPE in a day		
	n (%)	Age mean (SD)	Sex, male	Adverse skin reactions	Working hours/week, mean (SD)	<3 h/day	3‐6 h/day	>6 h/day
**All**	10 287	44.8 (12.4)	1433 (13.9)	6372 (61.9)	34.9 (5.6)	692 (6.7)	2891 (28.1)	6704 (65.2)
**Nurses**	5924 (57.6)	44.2 (12.4)	311 (5.2)	4057 (68.5)	34.1 (5.1)	289 (4.9)	1377 (23.2)	4258 (71.9)
**Nurse assistants**	800 (7.8)	52.1 (11.3)	47 (5.9)	509 (63.6)	32.7 (5.9)	31 (3.9)	165 (20.6)	604 (75.5)
**Physicians**	2330 (22.6)	45.3 (12.1)	890 (38.2)	996 (42.7)	38.0 (5.5)	312 (13.4)	930 (39.9)	1088 (46.7)
**Physiotherapists**	376 (3.7)	42.4 (11.9)	84 (22.3)	225 (59.8)	34.9 (4.3)	21 (5.6)	223 (59.3)	132 (35.1)
**Occupational therapists**	131 (1.3)	39.4 (11.0)	14 (10.7)	90 (68.7)	35.4 (3.5)	6 (4.6)	59 (45.0)	66 (50.4)
**Midwives**	351 (3.4)	42.1 (12.7)	2 (0.6)	249 (70.9)	32.1 (5.6)	13 (3.7)	28 (8.0)	310 (88.3)
**Radiographers**	375 (3.6)	42.6 (12.0)	85 (22.7)	246 (65.6)	35.6 (4.8)	20 (5.3)	109 (29.1)	246 (65.6)
Pearson's chi‐square test, *P‐*value			.001	<.001		<.001		
ANOVA, *P‐*value		<.001			<.001			

Abbreviations: ANOVA, analysis of variance; F‐PPE, face personal protective equipment; SD, standard deviation.

**TABLE 2:** Adverse skin reactions (ASRs) related to departments and different types of masks, n = 10 287, n (%)

	F‐PPE ≥1 types		Surgical masks		FFP3	
	used, n = 10 287	With ASRs, n = 6372	used, n = 9946	With ASRs, n = 5910	used, n = 4735	With ASRs n = 2156
In‐patient care	4350	2951 (67.8)	4201	2756 (65.6)	2352	1076 (45.7)
Outpatient clinic incl., for example, radiotherapy	3748	2016 (53.8)	3578	1890 (52.8)	1042	417 (40.0)
Operating theatre	791	436 (55.1)	786	411 (52.3)	371	124 (33.4)
Intensive care unit	716	540 (75.4)	708	468 (66.1)	511	330 (64.6)
Department of anesthesiology	509	305 (59.9)	505	269 (53.3)	384	173 (45.1)
Recovery room	173	124 (71.7)	168	116 (69.0)	75	36 (48.0)
*P‐*value		<.001^a^		<.001^a^		<.001^a^

Abbreviation: F‐PPE, face personal protective equipment.

*Note*: % with ASRs in the specific departments.

^a^ statistically significant.

On a working day, HCWs used one or more F‐PPE (surgical masks [n = 9946], FFP3 [n = 4735], face shield [n = 6342], and/or goggles [n = 4955]) (Tables 2 and 3). Approximately 50% of them used both surgical masks and FFP3 (n = 4735), whereas others used only surgical masks (n = 1889), FFP3 (n = 61), goggles (n = 12), or face shields (n = 97).

**TABLE 3:** Different kinds of F‐PPE and associated adverse skin reactions (ASR) and skin symptoms, n = 10 287, n (%)

	Surgical masks	FFP3	Face shields	Goggles
Used, n	9946 (96.7)^a^	4735 (46.0)^a^	6342 (61.7)^a^	4955 (48.2)^a^
Used without ASRs	4036 (40.6)^b^	2579 (54.5)^b^	5230 (82.5)^b^	4644 (93.7)^b^
Used with ASRs	5910 (59.4)^b^	2156 (45.5)^b^	1112 (17.5)^b^	311 (6.3)^b^
**Use F‐PPE in h/day**				
<3 h	352 (32.8)^b^	1042 (48.3)^b^	357 (32.1)^b^	105 (33.8)^b^
Between 3 and 6 h	1696 (32.7)^b^	721 (33.4)^b^	448 (40.3)^b^	113 (36.3)^b^
>6 h	3862 (44.9)^b^	393 (18.2)^b^	307 (27.6)^b^	93 (29.9)^b^
*P*‐value = differences between use of hours of the F‐PPE	<.001^c^	<.001^c^	<.001^c^	<.001^c^
Greasy or sticky skin	1038 (10.4)^b^	313 (6.6)^b^	187 (2.8)^b^	27 (0.5)^b^
Itchy skin	2911 (29.3)^b^	1037 (21.9)^b^	485 (7.6)^b^	48 (1.0)^b^
Dry or scaly skin	2134 (21.5)^b^	654 (13.8)^b^	186 (2.9)^b^	31 (0.6)^b^
Red and irritated skin	3518 (35.4)^b^	1293 (27.3)^b^	482 (4.6)^b^	80 (1.6)^b^
Swollen and irritated eye area	759 (7.6)^b^	302 (6.4)^b^	82 (1.3)^b^	45 (0.9)^b^
Spots or pimples	3697(37.2)^b^	1013 (21.4)^b^	339 (5.3)^b^	39 (0.8)^b^
Tender, burning, or painful skin	1032 (10.4)^b^	434 (9.2)^b^	116 (1.8)^b^	31 (0.6)^b^
Fissures	351 (3.5)^b^	94 (2.0)^b^	23 (0.4)^b^	7 (0.1)^b^
Macerated and exuding skin	83 (0.8)^b^	36 (0.8)^b^	16 (0.3)^b^	1 (0.0)^b^
Pressure mark	1254 (12.6)^b^	1125 (23.8)^b^	456 (7.2)^b^	203 (2.0)^b^
Pressure ulcer	167 (1.7)^b^	126 (2.7)^b^	36 (0.6)^b^	26 (0.3)^b^

Abbreviations: F‐PPE, face personal protective equipment.

^a^ by total population, n = 10 287.

^b^ by those, who use the specific F‐PPE.

c statistically significant.

Of the study population, 61.9% self‐reported ASRs of the face related to the use of one or more types of F‐PPE (Table 1). The ASRs included itchy skin, dry or scaly skin, red and irritated skin, greasy or sticky skin, swollen and irritable eye area, spot or pimples, tender skin, burning or painful skin, fissures, macerated and exuding skin, pressure marks, and ulcers (Table 3).

The sites of ASRs included the area around the mouth (n = 3583), cheeks (n = 3541), chin (n = 3419), nose (n = 3042), ears (n = 1191), eyes (n = 976), forehead (n = 732), and neck (n = 779).

In the six hospitals, there were no statistically significant differences in the frequency of ASRs (*P* = .40). The frequency of ASRs was related to the department where the HCWs worked (Table 2). For example, the frequency of ASRs was significantly higher in intensive care units (F‐PPE, 75.4%; surgical masks, 66.1%; and FFP3, 64.6%) than in the department of anesthesiology (F‐PPE, 59.9%; surgical masks, 53.3%; FFP3, 45.1%) (*P* < .001).

### 3.1 Sex

The frequency of ASRs was significantly higher in women (n = 5832; 65.9%) than in men (n = 540; 37.7%) (*P* < .001). Some female HCWs (n = 3573; 40.4%) applied make‐up under the F‐PPE. However, there was no significant difference in the frequency of ASRs between women who did (n = 2328; 65.2%) and did not (n = 3504; 66.4%) apply make‐up (*P* = .25).

Men with beards (n = 694) reported significantly more ASRs (n = 285; 41.1%) than those without (n = 255; 34.5%) (*P* = .012).

### 3.2 Allergies

Nearly one third of HCWs had allergies (n = 3046; 29.6%) to pollen (n = 1960;19.1%), house dust mites (n = 1099; 10.7%), furred animals (n = 889; 8.6%), nickel (n = 674; 6.6%), perfume (n = 465; 4.5%), adhesive tape (n = 234; 2.3%), and latex (n = 208; 2.0%). HCWs with allergies reported ASRs significantly more frequently (n = 1972; 64.7%) than HCWs without allergies (n = 1074; 60.8%) (*P* < .001).

### 3.3 Professions

Physicians reported significantly less ASRs (42.7%) than nurses (68.5%), nurse assistants (63.6%), physiotherapists (59.8%), occupational therapists (68.7%), midwives (70.9%), and radiographers (65.6%) (*P* < .001) (Table 1).

### 3.4 Duration of F‐PPE use

The study population worked for a mean of 35 hours per week (standard deviation: 5.6). This is the typical number of working hours for HCWs in Denmark (Table 1). Most respondents used F‐PPE daily (85.4%), followed by 3‐4 days per week (11.3%), and 1‐2 days per week (3.3%). F‐PPE was worn for >6 hours per day (65.2%), followed by 3‐6 hours per day (28.1%), and < 3 hours per day (6.7%). The differences in frequencies of ASRs were associated with the duration of F‐PPE use. ASR differences between F‐PPE use >6 hours per day (n = 4585; 69.9%), 3‐6 hours per day (n = 1520; 52.6%), and < 3 hours per day (n = 167; 24.1%) were statistically significant (*P* < .001).

### 3.5 Chronic skin diseases

One fifth of respondents (19.1%) reported chronic skin diseases, such as atopic dermatitis (5.4%), rosacea (4.0%), psoriasis (3.9%), acne (3.7%), seborrheic dermatitis (1.4%), and other skin diseases (hand eczema [n = 308], urticaria [n = 32], periorbital dermatitis [n = 41], and perioral dermatitis [n = 41]) (Table 5). The proportion of HCWs who received treatment for chronic skin diseases was 7.6% (n = 778). Treatments included topical corticosteroids (n = 582), tacrolimus (n = 41), ketoconazole (n = 17), antihistamines (n = 29), and various treatments for acne and rosacea (n = 178).

The frequency of ASRs was significantly higher in HCWs with chronic skin diseases (n = 1407; 71.6%) than in those without (n = 4965; 59.7%) (*P* < . 001). This was also true for acne (84.2% vs 61.1%; *P* = .001), atopic dermatitis (72.3% vs 61.3%; *P* < .001), and rosacea (79.6% vs 61.2%; *P* < .001), but not for psoriasis (66.5% vs 61.8%; *P* = .10) (Tables 4 and 5).

**TABLE 4:** Chronic skin diseases, adverse skin reactions (ASRs), and skin symptoms related to F‐PPE use, n = 10 287 with ASRs, n (%)

	Total	Acne			AD			Rosacea			Psoriasis		
	10 287	With acne = 385	Without acne n = 9902	*P‐*value	With AD n = 560	Without AD n = 9727	*P‐*value	With rosacea n = 407	Without rosacea n = 9880	*P*‐value	With psoriasis n = 319	Without psoriasis n = 9968	P‐value
ASRs related to F‐PPE	6372 (61.9)	324 (84.2)	6048 (61.1)	<.001*	405 (72.3)	5967 (61.3)	<.001*	324 (79.6)	6048 (61.2)	<.001*	212 (66.5)	6160 (61.8)	.101
Itchiness	3356 (32.6)	174 (45.2)	3182 (32.1)	<.001*	245 (43.8)	3111 (32.0)	<.001*	153 (37.6)	3203 (32.4)	.031*	117 (36.7)	3239 (32.5)	.129
Dry or scaly skin	2359 (22.9)	121 (31.4)	2238 (22.6)	<.001*	193 (34.5)	2166 (22.3)	<.001*	88 (21.6)	2271 (23.0)	.548	58 (40.0)	2301 (22.7)	<.001*
Red and irritated skin	4005 (38.9)	219 (56.9)	3786 (38.2)	<.001*	303 (54.1)	3702 (38.1)	<.001*	258 (63.4)	3747 (37.9)	<.001*	147 (46.1)	3858 (38.7)	.009*
Spot or pimples	3894 (37.9)	293 (76.1)	3601 (36.4)	<.001*	213 (38.0)	3681 (37.8)	.929	206 (50.6)	3688 (37.3)	<.001*	122 (38.2)	3772 (37.8)	.907
Tender, burning, or painful skin	1278 (12.4)	50 (13.0)	1228 (12.4)	.753	114 (20.4)	1164 (12.0)	<.001*	81 (19.9)	1197 (12.1)	<.001*	50 (15.7)	1228 (12.3)	.084
Greasy or sticky skin	1210 (11.8)	104 (27.0)	1106 (11.2)	<.001*	69 (12.3)	1141 (11.7)	.686	50 (12.3)	1160 (11.7)	.753	40 (12.5)	1170 (11.7)	.724
Pressure marks	2107 (20.5)	112 (29.1)	1995 (20.1)	<.001*	117 (20.9)	1990 (20.5)	.829	94 (23.1)	2013 (20.4)	.188	65 (20.4)	2042 (20.5)	.995

Abbreviations: AD, atopic dermatitis; F‐PPE, face personal protective equipment.

*Note*: With = n (%) ASRs related to the specific chronic skin diseases. Without = n (%) ASRs related to no specific chronic skin disease.

* statistically significant.

**TABLE 5:** Relationship of skin types to adverse skin reactions (ASRs), chronic skin diseases, and F‐PPE use, n = 10 287, n (%)

	Total population	ASRs	Chronic skin diseases	Acne	Atopic dermatitis	Rosacea	Psoriasis
n (%)	n = 10 287 (100)^a^	n = 6372 (61.9)^a^	n = 1966 (19.1)^a^	n = 385 (3.7)^a^	n = 560 (5.4)^a^	n = 407 (4.0)^a^	n = 319 (3.9)^a^
Skin type				**n (%) of skin type)**			
Dry	5263 (51.2)^a^	2857 (44.8)^b^	715 (36.4)^c^	62 (16.1)^c^	224 (40.0)^c^	98 (24.1)^c^	159 (49.8)^c^
Oily	986 (9.6)^a^	592 (9.3)^b^	114 (5.8)^c^	48 (12.5)^c^	2 (0.4)^c^	22 (5.4)^c^	15 (4.7)^c^
Combination	1998 (19.4)^a^	1462 (22.9)^b^	393 (20.0)^c^	171 (44.4)^c^	61 (10.9)^c^	78 (19.2)^c^	59 (18.5)^c^
Sensitive	1453 (14.1)^a^	1145 (18.0)^b^	653 (33.2)^c^	83 (21.6)^c^	255 (45.5)^c^	195 (47.9)^c^	71 (22.3)^c^
Unknown	587 (5.7)^a^	316 (5.0)^b^	91 (4.6)^c^	21 (5.5)^c^	18 (3.2)^c^	14 (3.4)^c^	15 (4.7)^c^
*P*‐value		<.001*	<.001*	<.001*	<.001*	<.001*	<.001*

Abbreviation: F‐PPE, face personal protective equipment.

^a^ % by total population, who used F‐PPE, 10.287.

^b^ % by those with ASRs.

^c^ % by those with specific skin disease.

* statistically significant.

### 3.6 Skin symptoms

ASRs were attributed to chronic skin diseases (Table 4). For example, HCWs with pre‐existing acne had significantly more skin symptoms such as spots and pimples (76.1%), red and irritated skin (56.9%), itch (45.2%), dry and scaly skin (31.4%), pressure marks (29.1%), and greasy and sticky skin (27.0%) than those without acne (*P* < .001) (Table 4).

Different types of ASRs were attributed to different types of F‐PPE used. For example, surgical masks were reported to result predominantly in spots or pimples (37.2%), whereas FFP3 were reported to predominantly cause red and irritated skin (27.3%) (Table 3).

### 3.7 Skin types

Regarding skin types, 5263 (51.2% of total population, n = 10 287) respondents reported dry skin, 1998 (19.4%) reported combination skin, 1453 (14.1%) sensitive skin, 986 (9.6%) oily skin, and 587 (5.7%) unknown skin type (Table 5). Some skin types were associated with a higher rate of ASRs related to F‐PPE use. Specifically, HCWs with sensitive (n = 1145; 78.8% of 1453 with sensitive skin) or combination (n = 1462; 73.2% of 1998 with combination skin) skin had significantly more ASRs than HCWs with dry (n = 2857; 54.3% of 5263 with dry skin) skin (*P* < .001). There seems to be a link between specific skin types and individual chronic skin diseases. The most common skin type with acne was combination skin (44.4%), with atopic dermatitis was sensitive skin (45.5%), with rosacea was sensitive skin (47.9%), and with psoriasis was dry skin (49.8%).

### 3.8 Prevention and counteractive methods

Nearly all the HCWs used one or more kinds of preventive or counteractive methods (94.2%). HCWs used moisturizers (79.7%) or different methods for cleaning their face, for example, with a cleanser (43.7%), water (40.7%), and/or soap and water (15.7%) (Table 6). Relatively few respondents protected their skin against pressure marks and ulcers by applying a foam dressing (1.8%) or silicone film (0.5%) (Table 6). Those who had ASRs significantly used preventive and counteractive methods more frequently.

**TABLE 6:** Preventive and counteractive methods for adverse skin reactions (ASRs), n = 10 287, n (%)

	n	With ASRs, n = 6372	Without ASRs, n = 3915	*P*‐value
Used ≥1 preventive or counteractive methods	9688 (94.2)	6254 (98.1)^a^	3434 (87.7)^b^	<.001^c^
Used moisturizer	8203 (79.7)	5655 (88.7)	2548 (65.1)^b^	<.001^c^
Cleaned with a cleanser	4491 (43.7)	3301 (51.8)^a^	1190 (30.4)^b^	<.001^c^
Washed with water	4185 (40.7)	2594 (40.7)^a^	1591 (40.6)^b^	0.943
Washed with water and soap	1612 (15.7)	946 (14.8)^a^	666 (17.0)^b^	.003^c^
Used topical steroids	444 (4.3)	416 (6.5)^a^	28 (0.7)^b^	<.001^c^
Used barrier cream	316 (3.1)	299 (4.7)^a^	17 (0.4)^b^	<.001^c^
Applied thin foam dressing	183 (1.8)	170 (2.7)^a^	13 (0.3)^b^	<.001^c^
Applied silicone tape/film	47 (0.5)	46 (0.7)^a^	1 (0.0)^b^	<.001^c^

^a^ by the population with ASRs, n = 6372.

^b^ by the population without ASRs, n = 3915.

c statistically significant.

HCWs who experienced ASRs (n = 6372) undertook different actions, such as visiting a doctor (n = 312; 4.9%), reporting the ASRs to the Labour Market Insurance (n = 200; 3.1%), performing other duties due to ASRs (n = 103; 1.6%), and absences due to illness caused by the ASRs (n = 35; 0.5%).

To estimate the combined risk of ASRs, a multiple binary logistic regression was performed, including the previously described parameters: sex, profession, skin type, chronic skin disease, allergy, number of working hours per week, hours per day with F‐PPE, days per week using F‐PPE, and the use of the individual F‐PPE. Because only allergy was not significant, many confounding factors for ASRs have to be considered in the risks of ASR (Table 7). We found that female HCWs have a higher risk of ASRs than male HCSs, and that nurses have a higher risk than physicians but lower risk than midwives. The HCWs using F‐PPE for >6 hours versus <3 hours per day had a four times higher ASR risk. The risk of ASRs is higher in HCWs who work in intensive care units and in‐patient clinics than in HCWs who work in other departments, signifying that longer working hours with prolonged use of facial protection increases the risk of ASRs.

**TABLE 7:** Risk estimation of adverse skin reactions using multiple binary logistic regression

Parameter		Odds ratio (95% CI)	*P*‐value
Sex	Female/male^a^	2.04 (1.78‐2.34)	<.001*
Professions	Nurses^a^	1^a^	
	Nurse assistants	0.84 (0.71‐0.99)	.033
	Physicians	0.52 (0.46‐0.59)	<.001*
	Physiotherapists	1^b^	.10
	Occupational therapists	1^b^	.90
	Midwives	1.39 (1.07‐1.80)	.013
	Radiographers	1^b^	.28
Department	Outpatient's clinic^a^	1^a^	
	Inpatient care	1.36 (1.22‐1.52)	<.001*
	Intensive care unit	1.77 (1.44‐2.17)	<.001*
	Anesthesiology	1^b^	0.37
	Recovery room	1^b^	0.089
	Operating theatre	1^b^	0.12
Chronic skin disease	Yes/no^a^	1.48 (1.31‐1.68)	<.001*
Skin type	Normal to dry^a^	1^a^	
	Normal to oily	1.29 (1.11‐1.50)	<.001*
	Combination	2.06 (1.83‐2.33)	<.001*
	Sensitive	2.63 (2.27‐3.06)	<.001*
	Unknown	1^b^	.13
Use of F‐PPE in a week	1‐2 days per week^a^	1^a^	
	3‐4 days per week	1.86 (1.39‐2.50)	<.001*
	Daily	2.25 (1.71‐2.97)	<.001*
Use of F‐PPE in h/day	<3 h^a^	1*	
	Between 3 and 6 h	2.62 (2.14‐3.22)	<.001*
	>6 h	4.12 (3.36‐5.05)	<.001*
Working hours per week	Risk per additional hour	1.012 (1.003‐1.021)	.007*
Used surgical masks	Yes/no^a^	2.63 (2.07‐3.34)	<.001*
Used FFP3	Yes/no^a^	1.21 (1.09‐1.33)	.008*
Used face shields	Yes/no^a^	1.18 (1.07‐1.29)	<.001*
Used goggles	Yes/no^a^	1.20 (1.09‐1.32)	<.001*

Abbreviation: CI, confidence interval.

^a^ Reference category where odds ratio is defined as 1.

^b^ Not significantly different from reference category.

* Statistically significant.

Table 7 shows that surgical masks have the highest risk, which was approximately double that of other F‐PPEs. The majority of the respondents primarily used surgical masks and only 3.3% of the HCWs did not use surgical masks. Each type of used F‐PPE, for example, surgical masks increases the risk of ASRs. Odds ratios should be multiplied to estimate the rate of ASRs after the use of more than one F‐PPE type.

## 4 DISCUSSION

The COVID‐19 outbreak is challenging for all HCWs at the collective and individual level. The concept of this study was based on HCWs’ self‐reported ASRs due to the extensive use of F‐PPE. Previous cross‐sectional surveys and case reports have described ASRs, with a prevalence of 35.0%‐97.0% in populations of 13 to 2315 participants.^1^, ^2^, ^5^, ^6^, ^7^, ^8^, ^9^, ^14^, ^19^ The present study included a much larger population of 10 287 HCWs, and an ASR prevalence at 61.9%.

In a study by Hua et Al, it is described how FFP3 is associated with more frequent ASR than surgical masks.^20^ The high frequency of ASRs attributed to FFP3 may be explained by the fit of the mask, which is tighter than surgical masks, and therefore generates more heat, resulting in higher skin hydration and sebum secretion.^3^, ^20^ However, in our study, we found that most ASRs were attributed to surgical masks rather than FFP3. This may be explained by the recommendation to generally use surgical masks in public indoor areas, and the use of FFP3 only in specific situations such as when caring for patients with COVID‐19.

HCWs employed in the anesthesiology department or operating theatre reported fewer ASRs than those in the intensive care unit, recovery room, and in‐patient care. This may be because HCWs at these departments used F‐PPE more frequently even before the COVID‐19 pandemic, and therefore, were more likely accustomed to apply prevention strategies such as regular breaks from wearing the F‐PPE throughout the working day.

ASRs were more common among nurses, who represented the largest group of participating HCWs in our study, than among physicians. However, midwives had the highest frequency of ASRs. In both groups, most respondents used F‐PPE for >6 hours per day. This may reflect the different types of contact with patients that nurses and midwives have compared with the types of contact with patients that physicians have.

The ASRs secondary to F‐PPE use may result from skin occlusion that subsequently leads to increased heat, higher skin hydration, increased trans‐epidermal water loss, elevated pH, and increased sebum secretion, along with the local pressure and friction from the close‐fitting masks, which may induce irritant contact dermatitis or worsening or flare‐up of endogenous dermatoses such as acne or rosacea.^3^, ^20^ Nearly 20% of respondents reported chronic skin diseases, and had the highest frequency of ASRs.

In line with other studies, atopic dermatitis was associated with self‐reported sensitive skin.^16^, ^17^ This study showed that HCWs with chronic acne mostly had combination or sensitive skin, whereas those with rosacea reported predominantly sensitive skin. The prevalence of ASRs may be related to the skin type. Experts recommend the use of moisturizers to prevent ASRs.^4^, ^11^, ^21^, ^22^, ^23^, ^24^, ^25^, ^26^, ^27^ However, it should be noted that the use of moisturizers on inflamed skin may be poorly tolerated and trigger flare‐ups that need to be treated.^22^ In this study, the most common preventive or counteractive method was applying a moisturizer. However, whether this intervention reduced the incidence of ASR is unknown. It is possible that some HCWs may have only used moisturizers once ASR occurred. Regarding prevention, experts most frequently recommend occasional breaks from using F‐PPE, improved hydration by applying moisturizers, and improving the design of these masks in regarding safety, comfort, and tolerability.^13^

Several experts recommend facial cleansing throughout each shift.^28^, ^29^ Approximately 44% of the HCWs in this study cleaned their face with a cleanser, and a small proportion cleaned their face with soap and water. A recent study showed that cleanser surfactants in soap can cause tightness, dryness, skin barrier damage, erythema, irritation, and itching immediately after cleansing.^30^ Experts suggest that cleansers with a neutral or acidic pH, close to the normal pH level of 5.5 of the stratum corneum, may be potentially less damaging to the skin than using soap, which has a pH of 10.0.^30^

Some experts recommend avoiding the application of make‐up under F‐PPE.^26^, ^28^ Make‐up may be an occlusive that acts as a trigger for ASRs for sensitive skin while using F‐PPE.^17^ In this study, there was no difference in the ASR frequency between women who did and did not use make‐up.

Very few HCWs in the current study (4.9%) and in that by Szepietowski et al (6.5%) sought medical consultation regarding their ASRs.^14^ A few work absences due to skin symptoms were noted in the study by Trepanowski et al (0.5%) and this study (0.5%).^15^ It is surprising that HCWs in our study, despite the high prevalence of ASRs (61.9%), did not visit a doctor. It is possible that some HCWs with pre‐existing skin conditions were already familiar with the management of their skin, or their symptoms were only mild or considered inevitable.

In this study, the questionnaire focused on facial skin. However, in the comments sections allotted in the questionnaire, nearly 400 respondents described reactions in their mucous membranes (eyes, nose, and mouth). These mucosal reactions were noted in another study of HCWs^13^; however, further investigation regarding the causes of these mucosal changes and prevention methods is required.^13^

This study had limitations. First, the survey was distributed to all employed clinical staff and yielded a response rate of 44.7%. Some professions may be poorly represented in our study. Second, this was a self‐report questionnaire and the ASRs, chronic skin diseases, and skin types were not validated by a dermatologist. Third, participants were asked to identify which F‐PPE they assumed resulted in the ASRs, but most participants often used several different types of F‐PPE throughout the day. Fourth, this study may have a recall bias because respondents reported ASRs during the previous 5 months irrespective of if they still were experiencing ASRs. Fifth, this study did not show the effects of the respondent's actions to prevent or treat ASRs. Finally, the frequency of ASRs (61.9%) may have been influenced by the winter season when this the study was conducted.^31^, ^32^ During the COVID‐19 pandemic, the clinical staff were offered F‐PPE of varying quality regarding its comfort and fit, which may have affected the frequency of ASRs.

## 5 CONCLUSIONS

This study showed that HCWs reported several ASRs related to the prolonged use of different types of F‐PPE. Those with chronic skin diseases and sensitive and combination skin types were more prone to ASRs. HCWs using F‐PPE for >6 hours versus <3 hours per day had a four times higher risk of ASRs. It is therefore important to consider chronic skin diseases and skin types in the prevention of F‐PPE‐related ASRs.

## CONFLICT OF INTEREST

The authors have no conflict of interest to declare with regard to the manuscript or its content. The authors received no financial support for this study.

### AUTHOR CONTRIBUTIONS

**Jette Grothe Skiveren:** Conceptualization (lead); data curation (lead); formal analysis (lead); funding acquisition (lead); investigation (lead); methodology (lead); project administration (lead); resources (lead); software (lead); supervision (lead); validation (lead); visualization (equal); writing – original draft (lead); writing – review and editing (lead). **Susan Bermark:** Conceptualization (equal); investigation (equal); methodology (equal); resources (equal); validation (equal); visualization (equal); writing – original draft (equal); writing – review and editing (equal). **Malene Folkersen Ryborg:** Conceptualization (equal); investigation (equal); methodology (equal); resources (equal); validation (equal); visualization (equal); writing – original draft (equal); writing – review and editing (equal). **Britt Nilausen:** Conceptualization (equal); investigation (equal); methodology (equal); resources (equal); validation (equal); visualization (equal); writing – original draft (equal); writing – review and editing (supporting). **Peter Alshede Philipsen:** Conceptualization (equal); data curation (equal); formal analysis (equal); investigation (equal); methodology (equal); resources (equal); software (equal); supervision (equal); validation (equal); visualization (equal); writing – original draft (equal); writing – review and editing (equal).

## Data Availability

The data that support the findings of this study are available from the corresponding author upon reasonable request.
